# Cross-Talk between Dnmt2-Dependent tRNA Methylation and Queuosine Modification

**DOI:** 10.3390/biom7010014

**Published:** 2017-02-10

**Authors:** Ann E. Ehrenhofer-Murray

**Affiliations:** Institut für Biologie, Humboldt-Universität zu Berlin, 10099 Berlin, Germany; ann.ehrenhofer-murray@hu-berlin.de; Tel.: +49-30-2093-49630

**Keywords:** Dnmt2, Pmt1, queuine, queuosine, translation, tRNA cleavage, epitranscriptomics, anticodon modification

## Abstract

Enzymes of the Dnmt2 family of methyltransferases have yielded a number of unexpected discoveries. The first surprise came more than ten years ago when it was realized that, rather than being DNA methyltransferases, Dnmt2 enzymes actually are transfer RNA (tRNA) methyltransferases for cytosine-5 methylation, foremost C38 (m^5^C38) of tRNA^Asp^. The second unanticipated finding was our recent discovery of a nutritional regulation of Dnmt2 in the fission yeast *Schizosaccharomyces pombe*. Significantly, the presence of the nucleotide queuosine in tRNA^Asp^ strongly stimulates Dnmt2 activity both in vivo and in vitro in *S. pombe*. Queuine, the respective base, is a hypermodified guanine analog that is synthesized from guanosine-5’-triphosphate (GTP) by bacteria. Interestingly, most eukaryotes have queuosine in their tRNA. However, they cannot synthesize it themselves, but rather salvage it from food or from gut microbes. The queuine obtained from these sources comes from the breakdown of tRNAs, where the queuine ultimately was synthesized by bacteria. Queuine thus has been termed a micronutrient. This review summarizes the current knowledge of Dnmt2 methylation and queuosine modification with respect to translation as well as the organismal consequences of the absence of these modifications. Models for the functional cooperation between these modifications and its wider implications are discussed.

## 1. tRNA Methylation by the Dnmt2 Family of RNA Methyltransferases

### 1.1. The Discovery of tRNA Methylation by Dnmt2

Dnmt2 enzymes were originally described as homologs of the DNA methyltransferases Dnmt1, Dnmt3a and Dnmt3b, which methylate position 5 of cytosine to create 5-methylcytosine (m^5^C) in DNA [1]. However, Dnmt2 homologs show at most a very weak in vitro DNA methylation activity, despite possessing all residues that are important for catalytic activity [2,3]. A major breakthrough came in 2006 with the discovery of robust methylation activity of Dnmt2 on tRNA^Asp^ [4], and the position of methylation was determined to be C38 (m^5^C38), which lies in the anticodon loop. Meanwhile, transfer RNA (tRNA) methylation has been described for the Dnmt2 homologs from human, mouse, *Drosophila melanogaster*, *Dictyostelium discoideum*, *Schizosaccharomyces pombe*, *Entamoeba histolytica* and *Geobacter sulfurreducens* (reviewed in [5]). With one exception [6], Dnmt2s methylate C38 of tRNA^Asp^, though some homologs also target tRNA^Glu^, tRNA^Gly^ and/or tRNA^Val^ (Table 1). Dnmt2 enzymes are strongly conserved throughout evolution [7], but are absent in *Saccharomyces cerevisiae*. The strong conservation suggests an important cellular function. However, the absence of Dnmt2 does not cause major defects in growth, development and fertility [5]. The biological role of Dnmt2s is described in more detail below.

### 1.2. Dnmt2 in the Fission Yeast Schizosaccharomyces pombe: In Vitro Activity

In 1995, before the description of tRNA methylation by Dnmt2, a DNA methyltransferase homolog was fortuitously discovered in the fission yeast *S. pombe* and was termed pombe methyltransferase 1 (Pmt1) [8]. Its existence was surprising at the time, because *S. pombe* was known to lack any detectable DNA methylation [9]. This lack of DNA methylation was attributed to the fact that *S. pombe* Dnmt2 carries a conspicuous deviation from the consensus sequence in catalytic motif IV of the enzyme. Most other homologs carry the sequence proline-proline-cysteine-glutamine (PPCQ), the cysteine being the residue that initiates the methylation reaction [3]. In *S. pombe*, this cysteine is preceded by a serine rather than a proline residue (PSCQ), and this “mutation” at the time was hypothesized to cause the enzyme to be inactive.

Motivated by the discovery of tRNA methylation for Dnmt2 enzymes [4], we reevaluated the in vitro activity of *S. pombe* Dnmt2 [10]. Perhaps not surprisingly, the recombinant enzyme shows robust in vitro methylation activity on C38 of tRNA^Asp^. It also methylates tRNA^Glu^ in vitro, but the activity is considerably weaker than on tRNA^Asp^. Thus, contrary to earlier speculations [11], the P to S mutation in the catalytic motif IV of *S. pombe* Dnmt2 does not inactivate the enzyme. Furthermore, mutation of the presumed catalytic cysteine residue of PSCQ (*pmt1-C81A*) abrogates enzymatic activity, indicating that this residue, as in its homologs, indeed is essential for catalytic activity of *S. pombe* Dnmt2 [10].

### 1.3. In Vivo Regulation of Dnmt2 in S. pombe by Nutritional Signals

In contrast to the in vitro analysis, our initial investigation of in vivo methylation by Dnmt2 in *S. pombe* was rather disappointing. In cells grown in standard complete or minimal medium, tRNA^Asp^ methylation as measured by RNA bisulfite sequencing was found to be rather low (approx. 14%–20% [10,12]). However, overexpression of the *pmt1^+^* gene under an inducible promoter yields 100% tRNA^Asp^ methylation, and tRNA^Glu^ methylation is marginally increased, indicating that Dnmt2 levels in wild-type (wt) cells are limiting for in vivo tRNA methylation.

The big surprise came when we cultivated *S. pombe* cells in growth medium for its sister model organism, the budding yeast *Saccharomyces cerevisiae*, naively assuming that “yeast is yeast”, and that the growth medium for one yeast would also be suitable for the other yeast. Remarkably, in *S. cerevisiae* growth medium, the tRNA^Asp^ methylation level in *S. pombe* increases to 100%, indicating that some difference in medium composition regulates Dnmt2 activity [10].

Standard *S. cerevisiae* full medium (yeast peptone dextrose, YPD) contains yeast extract (1% *w*/*v*), peptone (2% *w*/*v*) and glucose (2% *w*/*v*), whereas *S. pombe* full medium (yeast extract with supplements, YES) consists of yeast extract (0.5% *w*/*v*), glucose (3% *w/v*) and the supplements adenine, histidine, leucine, uracil and lysine (225 mg/L each). A closer investigation of all medium components revealed that the addition of peptone stimulates m^5^C38 methylation on tRNA^Asp^ to 100% [10]. Peptone is present in the *S. cerevisiae*, but not the *S. pombe* medium and is a complex mixture of nutrients, including vitamins, amino acids, peptides and many more constituents, which left us with the herculean task of reducing this complexity to a single causative component.

### 1.4. Stimulation of Dnmt2 Activity by Prior Queuine Incorporation at G34 of tRNA^Asp^

**Figure 1:**
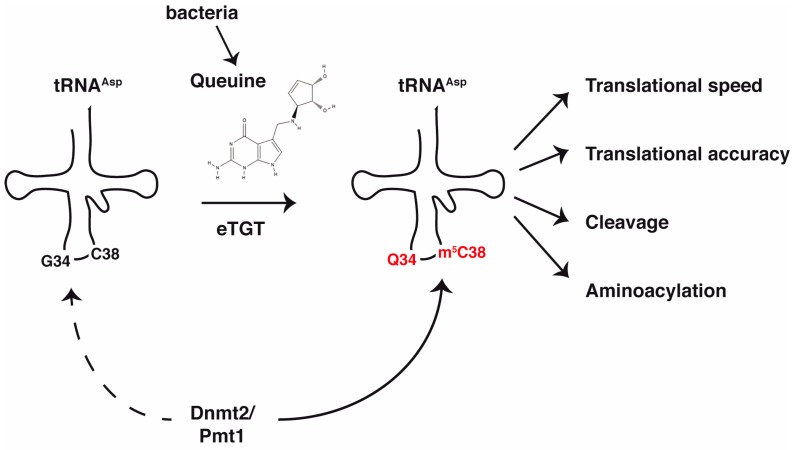
Stimulation of Dnmt2 activity in *S. pombe* (Pmt1) by prior Q incorporation. Dnmt2/Pmt1 shows low activity on non-Q-modified tRNA^Asp^ (dashed arrow), but is highly active on Q-modified tRNA^Asp^. Right, possible consequences of dual Q and m^5^C38 modification. eTGT: eukaryotic TGT enzyme.

In the course of identifying a stimulatory effect of peptone on Dnmt2-dependent tRNA methylation in vivo, we became aware of the fact that peptone, among others, contains the micronutrient queuine, which is incorporated at the G34 position of tRNA^Asp^ (see below, [13]). As kind of a “shot in the dark”, we therefore hypothesized that queuine might be the agent that, when introduced at G34 of tRNA^Asp^, stimulates Dnmt2-dependent tRNA methylation. Lo and behold, cultivating *S. pombe* in the presence of queuine resulted in an increase of in vivo tRNA^Asp^ methylation to 100% [12]. This revealed an unanticipated link between queuine modification and Dnmt2-dependent C38 methylation (Figure 1).

Further investigation of this link showed that the in vivo queuosinylation of the tRNA is necessary to stimulate *S. pombe* Dnmt2, since abrogation of queuine incorporation by deletion of the *S. pombe* genes encoding the enzyme responsible for the guanine–queuine exchange abolished in vivo stimulation of Dnmt2 by queuine.

### 1.5. Queuine-Independent Stimulation of Dnmt2-Mediated tRNA Methylation Is Mediated by Kinase Signaling

Apart from the queuine stimulation, we also identified queuine-independent means of increasing in vivo m^5^C38 methylation on tRNA^Asp^ in *S. pombe*. Specifically, cultivation of cells in medium with low ammonium levels also lead to increased in vivo methylation, whereas other conditions like low glucose levels, growth on non-fermentable carbon sources, or oxidative stress did not induce tRNA methylation. Interestingly, this stimulation required the function of the signaling kinase Sck2 [10]. One possibility is that Sck2 directly phosphorylates Dnmt2, and that the phosphorylated form has higher in vivo activity. Another possibility is that nutritional signals (e.g., nitrogen limitation) activate Sck2, which by a phosphorylation cascade leads to increased expression of Pmt1/Dnmt2, which in turn results in higher in vivo methylation levels.

## 2. Queuosine Modification on tRNAs

### 2.1. Queuine—Synthesis in Bacteria

Queuine is one of the most complex known RNA modifications [14]. It is a hypermodified guanine analog that comprises a 7-deaza-guanine core, to which an amino-methyl side chain and a cyclopentanediol moiety are attached (Figure 1) (reviewed in [13]). Queuine designates the base (q), and the respective nucleotide has been termed queuosine (Q). Queuosine is synthesized by eubacteria from guanosine-5′-triphosphate (GTP) in an elaborate biosynthetic pathway [15]. In a first set of enzymatic reactions, GTP is converted to the precursor preQ_1_ [16], which is then introduced into the wobble position of target tRNA molecules by bacterial tRNA guanine transglycosylases (bTGT) [17,18]. The preQ_1_ is then modified on the tRNA molecule in two further steps to generate the final queuosine product [19,20].

The substrates for queuosine modification are tRNAs with a GUN anticodon, i.e., tRNA^Asp^, tRNA^Tyr^, tRNA^His^ and tRNA^Asn^ [14]. As a result of the biosynthetic steps, guanine 34 (G34), which is in the wobble position of the anticodon, is replaced by Q.

### 2.2. Queuosine Modifications in Eukaryotes

Interestingly, Q in tRNA is not only found in bacteria, but is also abundant in eukaryotes (with the exception of *S. cerevisiae*, among others) [14]. However, contrary to bacteria, eukaryotes lack the enzymatic pathway for queuosine synthesis. Instead, they acquire queuine and queuosine from external sources like nutrition and from the gut microbiota [21,22,23,24] (Figure 1). Q-containing tRNAs from these sources as well as endogenous tRNAs are digested, resulting in queuosine nucleoside, queuosine-3′-monophosphate and free queuine base, which circulates e.g., in blood (serum) and other body fluids (milk, amniotic fluid) and is taken up by the cells [13]. Which transporter(s) are responsible for this uptake is not known. Q salvage in *S. pombe* involves a protein of the DUF2419 family whose homology suggests a role as a ribonucleoside hydrolase [25].

Queuine that has been taken up by the cell is then directly used by eukaryotic TGT enzymes (eTGTs) to exchange G for Q in the same group of tRNAs as in bacteria, namely those with a GUN anticodon (Table 1) [26]. Q modification is observed both on the cytosolic and the mitochondrial variant of these tRNAs [13,27]. The eTGTs consist of two subunits that both are evolutionarily related to bTGT: the catalytic subunit queuine-tRNA-ribosyltransferase (QTRT1) and the accessory subunit queuine-tRNA ribosyltransferase domain containing 1 (QTRTD1, re-annotated as QTRT2) [28]. It is interesting to note that eTGT acts as a heterodimer, whereas bTGT functions as a homodimer [29]. In addition, although QTRT1 and QTRTD1 are related to bTGT, QTRTD1 apparently has lost its enzymatic activity, but still is essential for eTGT activity. Further changes in enzymatic activity during evolution are seen by the fact that bTGT uses the precursor preQ_1_ as a substrate, whereas eTGT has evolved to exchange queuine or guanine, but not preQ_1_ [30].

In mammals, some Q-modified tRNAs can be subjected to further modification. The Q group on tRNA^Asp^ is further converted to mannosyl-Q (manQ), and the Q on tRNA^Tyr^ is converted to galactosyl-Q (galQ), whereas tRNA^Asn^ and tRNA^His^ are not further modified [31]. The enzymes responsible for this have not been isolated yet. Of note, in a similar mode, Q on tRNA^Asp^ in bacteria is modified to glutamyl-Q by the glutamyl-queuosine tRNA(Asp) synthetase yadB [32], whose activity requires the C38 position on the tRNA for glutamylation [33].

Of further interest is the fact that archaea also synthesize a 7-deazaguanine derivative, archaeosine (G^+^), which is incorporated at position 15 (in the D-loop) of most archaeal tRNAs with a G at this position. However, eTGTs cannot use archaeosine as a substrate [34].

## 3. Molecular Consequences of Queuosine Modification and Dnmt2-Dependent tRNA Methylation

### 3.1. Effect of Queuosine on the Dnmt2 Enzyme

Our investigation of Dnmt2-dependent m^5^C38 methylation of tRNA^Asp^ led us to discover an unexpected link between queuosine modification and methylation [12]. We found increased activity of Dnmt2/Pmt1 on tRNA isolated ex vivo that was Q-modified as compared to non-Q-containing tRNA. It will be interesting to determine how the presence of Q in tRNA stimulates Dnmt2 activity. The crystal structure of several Dnmt2 homologs has been determined, but unfortunately only without the tRNA substrate [35,36,37]. However, in one study, information on the effect of point mutations in Dnmt2 on tRNA binding and enzymatic activity was used to model tRNA^Asp^ binding on the Dnmt2 structure [38]. This suggests that Dnmt2 mainly contacts the anticodon loop and the stem of the tRNA in order to place the target cytosine near the active site of the enzyme. Dnmt2 induces an energetically unfavorable conformational change in the tRNA as it approaches the transition state. Thus, Q in the anticodon loop may alter the interaction of Dnmt2 with the substrate to improve energetics of the transition state, thus enhancing enzymatic activity. It is also conceivable that the presence of Q in the anticodon changes the geometry of the anticodon loop such that C38 is rotated more easily around the phosphodiester backbone and thus is more easily accessible for the catalytic cysteine residue of Dnmt2 and the cofactor.

### 3.2. Influence of m^5^C38 Methylation and Queuosine Modification on Translation

In considering the biological function of concomitant Q and m^5^C38 modification of tRNA^Asp^, the most obvious hypothesis is that these modifications affect codon-anticodon recognition and translation, since both modifications lie in the anticodon loop, and G34/Q34 is directly involved in base pairing with the codon in the messenger RNA (mRNA).

**Figure 2:**
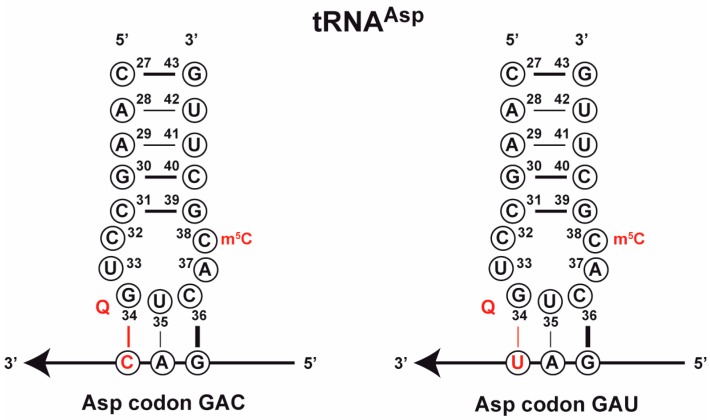
tRNA^Asp^ with the GUC (left) or QUC (right) anticodon decodes both aspartate codons, GAC (left) and GAU (right). The sequence of the anticodon stem-loop of tRNA^Asp^ from *S. pombe* is given.

**Figure 3:**
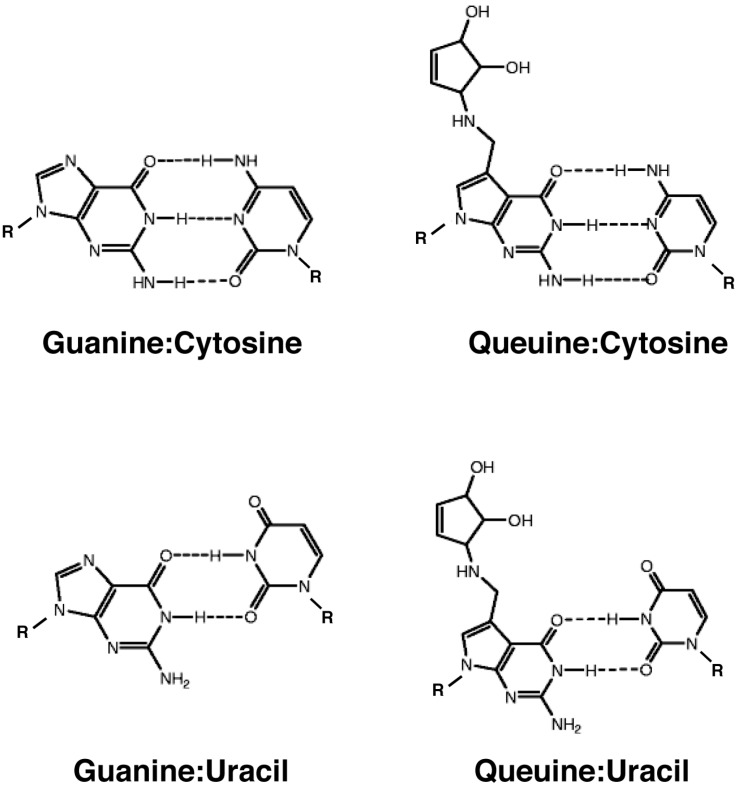
Base pairing between guanine, queuine and cytosine or uracil. Watson-Crick base pairing (top) and wobble base pairing (bottom) are shown. R: ribose.

Aspartate is encoded by two codons, GAC and GAU, which differ in the codon wobble base. However, the respective tRNA^Asp^ genes encoded in the majority of genomes only carry the anticodon GUC, which has Watson-Crick base pairing with the aspartate codon GAC in mRNA (Figure 2). For instance, *Escherichia coli*, *Schizosaccharomyces pombe*, *D. melanogaster* and mice exclusively have GUC anticodon tRNA^Asp^ genes (3, 8, 14 and 16 genes, respectively), and humans have 17 GUC genes, but only one AUC tRNA^Asp^ gene [39]. Nonetheless, both the GAC and the GAU codon are used in the genomes of these organisms (albeit in different ratios), and the GUC tRNA^Asp^ thus is used to decode both codons (Figure 3).

Of note, this situation of two codons encoding one amino acid, but only (or the majority of) tRNAs complementary to the C-ending codon existing in the genome also holds true for the other Q-containing tRNAs encoding asparagine (AAC, AAU), tyrosine (UAC, UAU) and histidine (CAC, CAU).

While G•U wobble base-pairing is common in RNA [40], a major question is whether Q and m^5^C38 affect this pairing and thus the ability of tRNA^Asp^ to read GAC versus GAU. There are multiple scenarios how differences in decoding can affect translation of proteins. If a modification reduces the speed of translation, this can increase ribosome pausing at a particular codon, which can cause defects in folding of the translated protein [41]. In addition, regulation of the entry of ribosomes into coding sequences can take place and can limit or exacerbate “traffic jams” of the ribosome [42]. Interestingly, in bacteria, there is a preference for the usage of certain codons in highly expressed genes [43,44,45]. Increased speed of a codon in the ribosome reduces the ribosomal density on a particular mRNA, leaving more unbound ribosomes for initiation and thus increasing overall protein production rates. Consequently, there is a selection for rapid elongation rates on highly expressed genes.

Besides speed, modification(s) influence the discrimination between cognate and near-cognate codons, i.e., translational accuracy. Misincorporation of amino acids into proteins can reduce or abrogate protein function and can cause cytotoxic misfolding. There thus is a major accuracy-driven selection during evolution for those codons that prevent errors in decoding, and the codon choice is influenced by tRNA modifications as well as by the abundance of the tRNAs [46]. The codons of the most abundant tRNAs are often assumed to be read more rapidly and more accurately than less abundant tRNAs, but misreading can also occur from highly abundant near-cognate tRNAs, thus reducing the accuracy of a particular codon (if it is more frequently misread than a synonymous codon) [47]. Hence, codon usage within a genome is a complex interplay of factors that act on translational speed and accuracy, which need to be taken into consideration when evaluating the effects of Dnmt2 and Q on translation.

#### 3.2.1. Effect on Codon-Anticodon Base Pairing

In a first approximation, Q shows the same ability as G to base-pair by hydrogen bonding with other bases, i.e., by forming three hydrogen bonds with C or two hydrogen bonds with U (Figure 3). Thus, one might expect that queuosine modification does not affect the ability of the tRNA^Asp^ to base-pair with the cognate aspartate codons. However, computational studies on the RNA bases concluded that queuine may have a slightly disfavored base pairing with cytosine as well as uracil [48]. This would suggest a weaker binding of Q than G and possibly a negative effect on codon-anticodon base pairing.

This modeling study is at odds with early work that measured the lifetime of anticodon-anticodon complexes in tRNA-tRNA interactions. Here, it was shown that the Q•U pairing increased stability of the complex by approx. 3-fold as compared to the same triplet containing G instead of U [49].

Of note, with the respect to the pairing of G with U, structural studies of this pair in the context of ribosome structures in states with incorrect tRNAs placed at the peptidyl-tRNA-binding site or the acceptor site (P-site and A-site) showed that G◦U mismatches at positions 1 and 2 of the codon mimic a canonical Watson-Crick geometry (i.e., three hydrogen bonds) through the formation of rare keto-enol tautomers of either uracil or guanine [50,51]. Interestingly, however, the G•U pair at the third position adopts the standard wobble geometry, possibly because of its larger degree of freedom at the tip of the U-turn fold of the anticodon loop.

Regarding the role of m^5^C38 in codon-anticodon pairing, since this position does not undergo direct contacts with the codon, in a first approximation, no direct effects on base pairing in the anticodon are expected.

#### 3.2.2. In Vivo Effects of Q and Dnmt2 on Translation

The effect of Q on codon preference within cells was measured in an early study in which G34- or Q34-tRNA^His^ was injected into Xenopus oocytes together with mRNA encoding the turnip yellow mosaic virus (TYMV) coat protein, and the incorporation of radiolabelled histidine at CAC and CAU histidine codons was measured [52]. It was observed that the unmodified tRNA had a preference for the C-ending codon, whereas the Q-modified tRNA showed equal decoding for the CAC and CAU codons. This led to the conclusion that Q incorporation affects the codon choice [52] and agrees with the anticodon-anticodon binding studies that reported the stabilization of complexes containing Q•U pairs [49].

Since the major target for methylation by Dnmt2 is tRNA^Asp^, one study hypothesized that the absence of Dnmt2 would be particularly detrimental for the translation of proteins carrying a poly-aspartate stretch [53]. Indeed, reporter constructs with an N-terminal stretch of six consecutive aspartates was less efficiently expressed in mouse MEF cells lacking Dnmt2. This observation was extended to several native proteins containing poly-aspartate stretches, which showed lower levels in the absence of Dnmt2, indicating that Dnmt2-mediated tRNA methylation is required for efficient translation of proteins containing poly-aspartate sequences [53].

A more comprehensive analysis of the genome-wide role of Dnmt2 in translation was performed by Tuorto et al. [54]. They used ribosome profiling to study Dnmt2 effects in mouse primary bone marrow cells. Importantly, the codons for the Dnmt2-modified tRNAs showed a reduction in ribosome density in the absence of Dnmt2. This was interpreted such that the lack of C38 methylation reduces the ability of tRNA^Asp^ to compete with near-cognate tRNAs and therefore allows more amino-acid misincorporation. In parallel, the study also measured differences in protein levels of wt and Dnmt2 mutant cells using two different methods. Among the down-regulated proteins, the authors observed a pausing of the ribosome on Asp codons in Dnmt2 mutant cells. This was interpreted to say that tRNA^Glu^, which has a wobble codon mismatch with the Asp codon, decodes Asp codons with a higher probability and thus causes miscoding. Altogether, this study concluded that m^5^C38 methylation by Dnmt2 serves to discriminate cognate codons from near-cognate codons [54].

#### 3.2.3. A Role for Queuine and Dnmt2 in Translational Fidelity

One major difference between guanine and queuine may come from the ability to distinguish allowed from disallowed base pairing at the wobble position in the translating ribosome and thus to prevent codon misreading. Especially the disallowance of guanosine-guanosine (G-G) pairing can involve a hydrogen bond between the N7 hydrogen of one G and the amino group of the other G [48]. This hydrogen bond is not possible with Q, since it lacks the 7-N position. Thus, the absence of queuosinylation could be expected to decrease the erroneous reading of the codon wobble base as G, which would result in glutamate incorporation into the peptide chain during translation. In this scenario, Q incorporation would thus reduce misreading a glutamate codon as aspartate and would increase translational fidelity.

**Figure 4:**
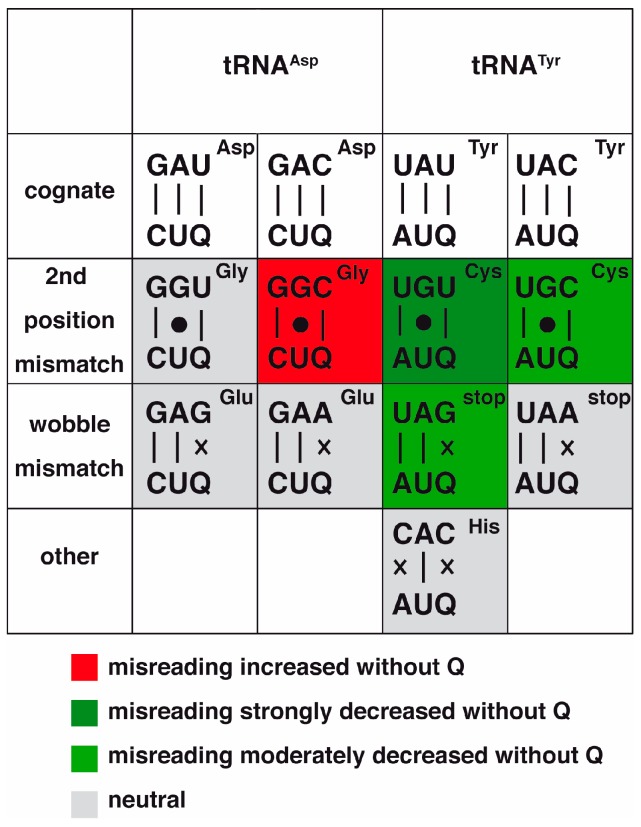
Effects of Q modification on mistranslation in bacteria [56]. Codon-anticodon pairs of tRNA^Asp^ and tRNA^Tyr^ are shown with the codon at the top and the anticodon at the bottom. The respective amino acid is indicated in the corner. First row, cognate codons for tRNA^Asp^ and tRNA^Tyr^. Second row, base mismatches at the second codon position that showed significant misreading in vivo [46]. Third row, wobble mismatches that showed significant misreading in vivo. Red color indicates that the codon showed increased misreading when the respective tRNA was not Q-modified. If the respective codon showed strongly or moderately decreased misreading in the absence of Q modification, the boxes are colored in dark green or light green, respectively. Grey, no effect of Q modification on misreading.

However, this view is contradicted by in vivo data investigating mistranslation of near-cognate codons in bacteria by tRNA^Asp^_QUC_ and tRNA^Tyr^_QUA_ [55]. Interestingly, both tRNAs showed a low misreading of near-cognate codons that differ in the wobble position (Glu codon GAA/GAG for tRNA^Asp^ and the stop codons UAA/UAG for tRNA^Tyr^) (Figure 4), and this was substantially lower than what has been observed for another tRNA, tRNA^Lys^_UUU_. It was thus hypothesized that this might be due to the Q modification that is absent from tRNA^Lys^. However, for tRNA^Asp^, the Q modification did not affect Glu codon misreading, showing that Q modification is not essential to block wobble misreading by tRNA^Asp^ of these codons [56]. In contrast, and contrary to expectation, for tRNA^Tyr^_QUA_, the absence of Q modification *lowered* rather than increased the mistranslation rates of the UAG stop codon, but had little effect on the UAA codon (Figure 4). Thus, apparently, the presence of Q modification increases translation error rates of tRNA^Tyr^_QUA_ that require a G•Q mismatch. This is contrary to the prediction made above regarding the Q reducing G-G base mispairing.

Both tRNA^Asp^_QUC_ and tRNA^Tyr^_QUA_ also showed some misreading of codons that differ in the second position. There was substantial misreading (1.6 × 10^−3^ per codon) of the glycine codons GGU and GGC by tRNA^Asp^_QUC_, and tRNA^Tyr^_QUA_ mistranslated the cysteine codons UGU and UGC (Figure 4). Both these mistranslations require a G◦U mispairing at the middle position [55], which, as mentioned above, has been shown to closely resemble the standard Watson-Crick geometry [50,51]. Importantly, the investigation of the role of Q modification in this scenario gave the surprising result that the error rate of tRNA^Asp^_QUC_ on the glycine GGC, but not GGU codon, was strongly *increased* in the absence of Q modification, showing that Q modification here served to *suppress* translation errors. In contrast, the middle position mismatching in both cysteine codons by tRNA^Tyr^ was *decreased* in the absence of Q modification, with the stronger effect being observed for the UGU codon, indicating that the modification actually *increases* error rates [56]. In short, the presence of Q decreased errors by tRNA^Asp^ and increased errors by tRNA^Tyr^. Thus, the effect of the modification on translation appears not to be dependent on the intrinsic properties of the modification itself, but rather seems to be sensitive to its structural context within the specific tRNA.

As a note of caution, it should be kept in mind that all these studies were performed in bacteria, where Q on tRNA^Asp^, but not tRNA^Tyr^, is further modified by addition of a glutamyl residue [57]. One explanation for different effects of Q on misreading by different tRNAs thus is that the glutamyl-Q has a different effect than Q alone. How these results translate to eukaryotic systems has not been determined.

Further insights into the role of Q modification in translational fidelity come from the structure of a ribosome with a C◦A mismatch between the CAC (histidine) mRNA codon and the Q-modified tRNA^Tyr^ (QUA) [51]. Here, the presence of Q at the first position led to the displacement of the mismatched cytosine at the third position from the codon-anticodon helix and to a distortion of the helix, thus enhancing the inability to form a stable C◦A interaction. In vivo, however, misreading rates of tRNA^Tyr^ on the CAC codon were slightly higher in the presence of Q modification [56], thus contradicting the assumption that Q modification would prevent misreading.

With respect to the role of Dnmt2 in translational fidelity, the study of Tuorto et al. concluded based on ribosome profiling that the absence of Dnmt2 increased wobble position misreading on aspartate and glutamate codons [54]. In agreement with this, they observed increased error frequencies, foremost with aspartate to glutamate and glutamate to aspartate misincorporations in proteins from Dnmt2 mutant cells, showing that Dnmt2-mediated tRNA methylation serves to suppress the misreading of near-cognate codons in mouse cells.

#### 3.2.4. Effect Q and m^5^C38 on the Architecture of the Anticodon Stem-Loop

Another hypothesis for the role of the Q and m^5^C38 modifications on tRNA is that they are part of the elaborate tRNA modification system whose role is to equalize the translational efficiency of all codons across the codon wheel. A priori, GC-rich anticodons are expected to show stronger base pairing with their respective codon, whereas AU-rich anticodons have weaker binding. If translation across all codons was to be kept more or less uniform across all codons, mechanisms must be in place to equalize between the different anticodons. A recent review by Grosjean and Westhof [58] proposed an alternative arrangement of the codon wheel that takes this into account and places GC-rich codons at the top of the wheel and AU-rich codons at the bottom. Their analysis of the anticodon loop suggests that codon-anticodon strength is influenced by the other positions in the anticodon stem as well as by positions other than the anticodon that lie in the loop. For instance, extension of the stem by base pairing of positions 32 and 38 is prevalent in GC-rich codons, suggesting that this serves to weaken the codon-anticodon interactions by imposing a strain on the anticodon loop. In this respect, it is interesting that tRNA^Asp^ has an unusual C-C pair at positions 32 and the methylated m^5^C38 position (Figure 2), and it can be speculated that this modification affects the stem-loop geometry.

A possible effect of m^5^C38 methylation in this context is to improve base stacking [59]. Bases 34 to 38 are stacked in the anticodon loop, and this stacking is potentially enhanced by m^5^C38, which would rigidify the anticodon loop.

In this regard, two Q-modified tRNAs (tRNA^Tyr^ and tRNA^Asn^) have “weak” anticodons (first two bases are AU-rich), and Q modification in these two can thus be hypothesized to render them “stronger” [58]. In contrast, tRNA^Asp^ and tRNA^His^ belong to the “intermediate” group (first two codon bases are a mixture of AU and GC), and Q modification may thus be expected to mildly weaken them. This could result in changes in residency time of the respective codons at the A-site of the ribosome and thus could affect translational speed.

#### 3.2.5. Effects of Q and m38C on tRNA Aminoacylation

The tRNA Q and m^5^C38 modifications also may affect aminoacylation of the tRNAs and thus influence translation by regulating the availability of tRNAs charged with the amino acid. Evidence for such a mechanism in relation to Q comes from two studies in the eighties that found a higher rate of aminoacylation for the Q than the G-containing equivalent [60], and the presence of Q slightly increased the affinity of bacterial tRNA^Tyr^ to tyrosyl-tRNA synthetase [17].

Interestingly, Dnmt2-dependent m^5^C38 caused a strong enhancement of aminoacylation by mouse aspartyl-tRNA synthetase. In vitro, a 5.5-fold increase in Vmax/Km for the methylated tRNA as compared to the unmethylated substrate was observed [53]. Furthermore, this translated to a 30% lower level of charged tRNA^Asp^ in vivo and indicated that the absence of m^5^C38 in cells leads to reduced availability of charged tRNA.

Since both Q and m^5^C38 modification enhance tRNA aminoacylation, one can speculate that the joint modification of tRNA^Asp^ would further enhance this affect and therefore would positively influence the availability of charged tRNAs for translation. How this affects translation in vivo remains to be determined.

#### 3.2.6. Affinity for the Ribosome

Another possible impact of Q and m^5^C38 methylation in translation is on the affinity of the tRNA for the ribosome. Higher affinity may be expected to reduce the accuracy of near-cognate codons due to misreading. Work in bacteria found that binding of Q-tRNA^Tyr^ to the ribosome was stimulated more strongly by UAU than by UAC, suggesting that Q enhanced the efficiency of binding of the tRNA to ribosomes [17]. Whether such an effect is seen in other organisms and how it impacts in translation in vivo is not clear, and whether a comparable effect is caused by Dnmt2-mediated tRNA methylation is not known.

#### 3.2.7. Protection of tRNAs from Endonucleolytic Cleavage by Q and m^5^C38 Modification

Certain tRNA modifications influence the ability of tRNAs to be cleaved in the anticodon loop by RNA endonucleases, and their cleavage thus can affect cellular levels of functional tRNAs that are available for translation (reviewed in [61]). tRNA cleavage can be executed by endogenous endonucleases, which may serve to regulate tRNA levels under stressful circumstances. Furthermore, tRNA cleavage is a strategy employed by some pathogens [62], e.g., by killer toxins of viruses that infect yeast cells [63,64], and thus, protection against cleavage may be a mechanism for pathogen defense.

Since both Q and m^5^C38 are in the anticodon loop, this seems to suggest that these modifications could affect tRNA cleavage. For Dnmt2-mediated tRNA methylation, increased cleavage of Dnmt2 tRNA substrates was observed in Dnmt2-mutant *Drosophila* flies that were subjected to oxidative stress [65], suggesting that the responsible endogenous RNAse(s) were induced under stress conditions, and that Dnmt2-mediated methylation protects against cleavage. This protection was recapitulated in vitro using the RNAse A-like ribonuclease angiogenin, which cleaved tRNAs from *Drosophila* and mice more readily when they were not methylated by Dnmt2 [65]. An intriguing possibility is that the resulting tRNA fragments have a specific function in vivo, and they are possibly related to the inheritance of epigenetic states (see below).

For Q modification, it remains to be seen whether Q-specific tRNA nucleases exist. Interestingly, the *E. coli* ribonuclease colicin E5, a plasmid-encoded killer toxin, specifically cleaves all potentially Q-modified tRNAs and hydrolyzes them between Q34/G34 and U35 in the anticodon. However, colicin E5 cleaves its targets irrespective of whether position 34 is Q-modified or not [66]. A structural study of the interaction between colicin E5 and a minimal substrate showed that the interaction with its substrate does not involve the N7 position of guanine which would differ in a Q-containing tRNA [67]. Hence, Q is not the determinant of substrate recognition by colicin E5. Thus, a situation like with the killer toxin zymocin from *Klyuveromyces lactis*, which only cleaves tRNAs that are modified at the wobble position by elongator [61], remains to be identified.

With respect to a possible function in the protection of nucleic acids by Q-related modifications, 7-deazaguanine derivatives were recently identified in the DNA of an isolate of *Salmonella enterica* as well as some other bacteria [68]. Interestingly, evidence was found for a restriction-modification system utilizing this modification, since DNA from a modification-deficient strain transformed into a modification-proficient strain caused a dramatic loss of transformation efficiency. It thus was speculated that DNA restriction enzymes exist in bacteria that can distinguish between unmodified DNA and DNA carrying Q-related modifications.

### 3.3. Evolutionary Conservation of the Dependence of Dnmt2 on Q Modification

Our discovery of a Q-mediated mechanism of C38 methylation by Dnmt2 in *S. pombe* [12] begs the question whether Dnmt2 homologs from other organisms are also sensitive to prior Q incorporation. Our use of *S. pombe* as a model system was fortunate, because media conditions can be easily be manipulated, which allowed us to discover this unexpected link. However, Q is omnipresent in other model systems under standard laboratory conditions (*Drosophila*, mice, human cells) [13], such that the Q-dependence of Dnmt2 for this reason may have gone unnoticed. Furthermore, the Q-dependence is not easy to evaluate in these systems, because rendering them Q-deficient requires complex experimental setups with fully synthetic media or nutrition, and in the case of mice, animals lacking gut microflora (axenic mice) must be used [22]. Standard cultivation medium for cell culture requires addition of serum (e.g., fetal calf serum), which contains copious amounts of queuine. One possibility to circumvent this is to test Dnmt2-dependent m^5^C38 methylation levels in tRNA from organisms lacking the tRNA guanine transglycosylase, but this remains to be done.

We have used the experimental system of *Dictyostelium discoideum* to test Q-dependence of a higher eukaryotic Dnmt2. Even though the standard growth conditions were not Q-free, adding further Q to the medium stimulated m^5^C38 methylation on tRNA^Asp^, suggesting some degree of Q-dependence in this system [12]. It will be crucial to test Q-dependence in other systems, foremost humans, and to see whether the two modifications jointly affect translation.

In this context, it is noteworthy that several model systems harbor both the Dnmt2 and Q modification system (Table 1), whereas *S. cerevisiae* (and closely related yeasts) carries neither system. This would argue in favor of an evolutionary co-dependence of the two systems. However, *C. elegans* (and most likely other related nematodes) display Q modification, but have no Dnmt2 homolog (Table 1). It will thus be interesting to determine whether Q modification in this Dnmt2-less context has another function than in Dnmt2-carrying organisms.

It still remains possible that the strong Q-dependence that we observe for *S. pombe* Dnmt2 is unique to (or strongest in) this organism. If this is the case, one possible reason for this is the sequence deviation of *S. pombe* Dnmt2 in the catalytic site as compared to its homologs [69]. The PSCQ sequence in catalytic motif IV is not unique to *S. pombe*, but is also found in a number of other filamentous fungi, while all larger eukaryotes harbor the PPCQ version. A subgroup of fungi carry yet another sequence variation, an alanine substitution at the second position (PACQ) (there is one organism, *Rozella allomycis*, that carries yet another version, PCCQ), and it will be interesting to see whether these versions affect the activity of the Dnmt2 protein with respect to its sensitivity to prior Q incorporation into the tRNA substrate.

## 4. Organismal Functions of Queuosinylation and C38 Methylation

### 4.1. Biological Consequences of Dnmt2-Mediated tRNA Methylation

#### 4.1.1. Dnmt2 Mutant Phenotypes in Mice: Roles in Translation and Epigenetic Inheritance

While Dnmt2 is highly conserved across evolution, implying an important functional role for this protein, its absence in most organisms does not cause gross morphological defects (reviewed in [70]). *S. pombe*, *Drosophila* and mice lacking Dnmt2 are viable and fertile. There is one notable exception to this, zebrafish, where a morpholino knock-down showed strong developmental defects [71], but this observation has not been independently evaluated. A more careful investigation of Dnmt2-lacking organisms reveals more subtle differences when compared to the wt counterparts, though the relationship of these phenotypes to the enzymatic activity of Dnmt2 is not always obvious.

To date, the strongest Dnmt2 mutant phenotype has been observed in mice, where the absence of both Dnmt2 and a second tRNA methyltransferase, NSun2, which generates m^5^C at other tRNA positions, causes embryonic lethality [72]. The complete absence of m^5^C in Dnmt2/Nsun2 double mutant cells caused reduced protein synthesis and reduced tRNA levels, which is consistent with a role of m^5^C in translation as well as in tRNA stability (which is regulated by cleavage). A closer inspection of Dnmt2 mutant mice revealed that they have a delay in endochondral ossification and a reduction in haematopoietic stem and progenitor cell populations [54]. Furthermore, mutant mice have cardiac hypertrophy, though cardiac function seems not to be disturbed. In embryonic stem cells, the absence of Dnmt2 was accompanied by increased activity of RNA polymerase II, which was attributed to decreased levels of non-coding RNAs that exert an inhibitory effect on RNA polymerase II. It was proposed that Dnmt2 methylates and stabilizes these RNAs. However, the methylation was determined using an anti-methylcytosine antibody and was not detectable using RNA bisulfite sequencing [73].

A further phenotype of the absence of Dnmt2 is that it influences the inheritance of an epiallele of the *Kit* gene. This allele (*Kit^tm1Alf^*) causes a white-tail phenotype in heterozygous *Kit^tm1Alf/+^* mice. Remarkably, the phenotype is inherited to *Kit^+/+^* offspring of these heterozygotes, i.e., the mice have the mutant phenotype despite not carrying the *Kit* epiallele [74]. This therefore is a paramutation-like phenomenon. Remarkably, the paramutant phenotype is not transmitted to the offspring of *Dnmt2^−/−^* mice. Furthermore, RNA extracted from paramutant animals and injected into fertilized wt eggs gives rise to paramutant offspring, invoking an RNA-mediated mechanism for the epigenetic inheritance. However, injection of RNA from Dnmt2^−/−^*Kit* paramutant animals did not induce paramutation, indicating that a Dnmt2-mediated RNA modification is required to produce the mutant phenotype. The exact mechanism for this phenomenon remains to be determined. The analysis of RNA methylation of the *Kit* mRNA was suggestive of a slight increase in methylation at two sites in wt, but not Dnmt2^−/−^ embryos, but it is not clear whether these represent deamination artifacts [75].

In this context, it is interesting to note that a link has been identified between tRNA halves and epigenetic inheritance. Specifically, the effects of paternal diets on the metabolism of the offspring are accompanied by increases in small RNAs, in particular the 5′ fragments of tRNAs (especially tRNA^Gly^), in the sperm of mice subjected to protein restriction [76,77], and it has been proposed that these RNA fragments affect gene expression in the offspring. In fact, injection of the 5′ tRNA^Gly^ fragment represses the expression of approximately 70 genes in the early embryo, including the murine endogenous retrovirus MERVL [77]. Thus, this tRNA fragment may have properties comparable to those of a microRNA. The involvement of such a tRNA fragment in the epigenetic inheritance of these nutritional effects resonates with the fact that Dnmt2 plays a role in paramutation in mice [75], and that it protects tRNAs from endonucleolytic cleavage. How these pieces of the puzzle are connected remains to be seen.

#### 4.1.2. Consequences of Absence of Dnmt2 in Flies: Transposon Silencing, Stress Resistance and Immune Control of Pathogens

As for mice, Dnmt2 mutant *Drosophila* lack obvious growth or developmental phenotypes [78]. However, using insertions of reporter genes into different heterochromatic regions in the fly genome, it was shown that Dnmt2 is required for silencing of *Invader4* retrotransposons, but not for pericentric heterochromatin silencing [79]. It is unclear how this phenotype relates to the enzymatic activity of Dnmt2, and effects on DNA methylation have been suggested [79] but disputed [80]. Dnmt2 mutant flies furthermore show increased viral load and have an activated innate immune response [81]. Conversely, Dnmt2 overexpression reduces infection of *Drosophila* with *Wolbachia* and reduces rates of cytoplasmic incompatibility caused by *Wolbachia* [82]. In addition, Dnmt2 was found to bind to viral RNA, though it is not known whether these RNAs are also methylated by Dnmt2 [83]. Heat shock of flies is accompanied by the appearance of tRNA fragments whose levels are increased in the absence of Dnmt2, showing, as in mice [65], a protective role for Dnmt2-mediated methylation against endonucleolytic cleavage. Furthermore, such tRNA fragments impair the function of Dicer enzymes that regulate small interfering RNA (siRNA) pathways [83]. Thus, one function of Dnmt2 enzymes may be to suppress aberrant tRNA fragmentation and thus to ensure the correct regulation of siRNA pathways under stressful conditions.

A further indication towards a role for Dnmt2 in the protection against adverse circumstances (heat shock, pathogens) comes from a study that reported an increase in the lifespan of flies upon Dnmt2 overexpression [84], though molecular insights into this phenotype of Dnmt2 are missing.

A less intuitive role for Dnmt2 in flies was found for the unequal segregation of sister chromatids during stem cell division. Specifically, it was observed that the sister chromatids of the X and Y chromosomes show an asymmetric inheritance during mitosis of male germline stem cells. Remarkably, this non-random segregation was abrogated in Dnmt2 mutants, indicating that Dnmt2 imparts some kind of “imprint” on the sex chromosomes during gametogenesis that leads to non-random segregation in the germline stem cells of the progeny [85]. How this works mechanistically is unclear, but is conceptually linked to the role of Dnmt2 in epigenetic inheritance in mice.

#### 4.1.3. Dnmt2 Mutant Phenotypes in Other Organisms

The observations in flies of a role of Dnmt2 in stress tolerance are reflected in phenotypes in the moss *Physcomitrella patens*, where Dnmt2 mutant plants were found to have a delayed recovery from salt or osmotic stress [86]. Here, Dnmt2 also regulated the levels of tRNA^Asp^ in normal as well as in stressed cells. Thus, this phenotype of stress sensitivity in Dnmt2 mutant organisms may be a general theme of Dnmt2 function. This may also hold true for those organisms for which a phenotype so far has not been found, possibly because the “right” stress conditions have not been identified yet. This is also the case for *S. pombe*, where we have looked for phenotypes of Dnmt2 mutants (*pmt1∆* strains) under conditions of oxidative, heat or nutritional stress, but found no growth differences to the wt strain [10], nor did we observe effects on heterochromatin silencing [87]. Thus, while Dnmt2 in such organisms has a clear “molecular” phenotype (i.e., loss of tRNA methylation), the functional consequences are not yet known in all systems.

Another common theme for Dnmt2 function may be retrotransposon silencing, since upregulation of expression was observed in *Drosophila* [79] and also in *Dictyolstelium*, where the increased transcription and mobilization of the Skipper retrotransposon was found [88]. The relationship between transposon silencing and RNA methylation is not clear.

### 4.2. Organismal Roles for tRNA Queuosinylation

While Dnmt2 mutant organisms have been heavily scrutinized for phenotypes [5], less phenotypic analysis has been performed for the effects of loss of Q modification [13]. We discovered the Q-dependence of Dnmt2 in *S. pombe*, yet cultivating cells in the presence of Q confers no obvious growth advantage, and in fact, standard growth medium for *S. pombe* lacks Q sources [12]. Obtaining larger eukaryotic organisms that are Q-free is challenging (see above), but has been done in the past. Q-free mice were obtained by feeding axenic (germ-free) mice a fully synthetic diet lacking Q. Such mice were reported to be asymptomatic [89], but they manifest strong phenotypes (ocular and neurological defects) and lethality when they additionally are deprived of tyrosine [90]. Tyrosine is a non-essential amino acid that is synthesized from phenylalanine by the phenylalanine hydroxylase, which requires tetrahydrobiopterin as a co-factor. Further analysis showed that Q depletion reduces tetrahydrobiopterin levels and thus restricts the biosynthesis of tyrosine (as well as that of dopamine, seratonin, and nitric oxide), and it was hypothesized that this co-factor reduction is the consequence of broad translational defects in the absence of Q [91]. Q deprivation caused no apparent phenotype in *Dictyostelium* [92], flies [93] and *Caenorhabditis elegans* [24].

An alternative way to study the role of Q is to investigate organisms lacking the enzyme incorporating Q into the tRNAs, TGT. The original work describing the discovery of the TGT gene in bacteria reported that the absence of TGT, and thus Q-modification of the respective tRNAs in bacteria, led to a slight growth advantage under standard growth conditions, but to a disadvantage under unsuitable conditions [17]. It thus can be hypothesized that Q incorporation is beneficial under natural environmental conditions that most likely are stressful to cells, which may mirror the situation for Dnmt2. Mice lacking TGT are viable and asymptomatic [91], which agrees with the absence of phenotypes of Q-deficient mice [89]. Of note, the TGT mutants were insensitive to reduced amounts of tyrosine, possibly because sufficient levels of tyrosine and queuine were provided by gut microbes in this experimental setup [91]. It is also possible that the absence of Q and the inability to incorporate Q into tRNA have distinct biological consequences.

A possible medical application for a Q-deficiency phenotype comes from the discovery that the *tgt* gene from *Shigell flexneri*, a bacterium that causes diarrhea in humans, is a virulence factor, i.e., mutation in *tgt* impairs the ability of *Shigella* to infect host cells [94]. The underlying cause is that TGT is required for the synthesis of the virulence factor virF [95]. Surprisingly, bTGT can modify *virF* mRNA in vitro [96], implying that Q-modification of the mRNA, rather than virF translation, plays a role in the pathogenicity, though it is not known whether this modification also takes place in vivo. These observations have been the basis for efforts to develop inhibitors of bTGT as novel antibacterial compounds for the treatment of shigellosis [97].

A further potential use of the Q pathway for medical purposes comes from the fact that eukaryotic TGT is rather promiscuous in its substrate specificity. A novel TGT substrate, NPPDAG (*N*-((2-amino-4-oxo-4,7-dihydro-3H-pyrrolo[2,3-d]pyrimidin-5-yl)methyl)-3-phenylpropan-1-aminium choride), was developed, and this compound was found to restore defects in an animal model of multiple sclerosis. The therapeutic effect entirely depends on the presence of TGT [98]. How the presumed incorporation of NPPDAG into tRNAs leads to the amelioration of the multiple sclerosis phenotypes is not known.

### 4.3. Combined Phenotypes for the Absence of Dnmt2 and Q

Whether the combination of lack of m^5^C38 tRNA methylation and Q modification of tRNAs results in an enhancement of phenotypes as compared to either defect alone remains to be seen. At least in *S. pombe*, there are no observable defects when both modifications are missing, but the situation in multicellular organisms most likely is more complex. The most feasible avenue towards such insights will be to generate *tgt dnmt2* double mutant animals and to investigate them for defects. In the simplest scenario, there will be an enhancement of defects, since two anticodon modifications are affected at once.

## 5. Q as a Micronutrient and C38 Modification

### 5.1. Variations in Q Levels during Development and in Different Organs

One of the most intriguing facts about the link between Q and Dnmt2 modification is that Q is omnipresent in almost all eukaryotes, but originally comes from bacterial sources and is introduced into eukaryotes via their food and the gut microbiome. Thus, apart from its own effect on tRNAs in translation and translational accuracy, Q has the additional effect of modulating Dnmt2-dependent methylation of C38 in tRNA^Asp^ and thus further modifying translation. This presents an interesting path for the environment and in a broader sense and the microbiome to exert an effect on translation in the host. Q levels are lower in highly proliferating tissues and in cancer cells (reviewed in [99]), and they fluctuate during development in *Drosophila* [100,101]. Thus, the Q-mediated effect on Dnmt2 may vary across developmental stages and in different organs, thus leading to a complex pattern of effects on a multicellular organism or during disease. It will be interesting to see whether this fluctuation of Q corresponds to altered Dnmt2-dependent tRNA methylation levels in different tissues and in cancer cells, since this has not been investigated yet in detail.

One can envision many situations in which this interplay would vary. For instance, different individuals may have differences in their microbiome composition and thus varying levels of Q, such that differences in the bioavailability of Q may subtly alter translation in the host. Another scenario is starvation or malnutrition, where Q sources are expected to drop dramatically and thus to exacerbate detrimental effects in the starved organism.

### 5.2. Evolutionary Implications of Q/Dnmt2 Cooperation; Q and Dnmt2 in Natural Selection

All available data so far indicate that the effect of Q and Dnmt2 on translation at the level of an individual protein is likely to be mild. Yet, if regarded across evolutionary time, mild effects on translation may well shape fitness of an organism and act on natural selection on an organism in its natural environment. Genomes present a bias in codon usage that has evolved over evolutionary time to optimize the expression of particular genes in specific phases of development. One study investigated codon usage in 12 *Drosophila* species and found an unexpected reversal in accuracy-driven codon usage for Q-affected codons among Drosophilids [101]. Specifically, in one group of *Drosophilae* (e.g., *Drosophila melanogaster*), evolutionarily conserved Asp, His, Asn and Tyr residues were preferentially encoded by the NAC codon, whereas in another group (e.g., *Drosophila virilis*), NAU was preferred for such residues. Most importantly, the preference for the C-ending codon correlated with high Q-levels in the tRNA of adult flies. Q levels vary across developmental stages in *Drosophila*; they are high in embryos, sharply decrease, concomitant with rapid growth and development of the larvae, and they rise again in adults [101,102]. This drop in Q-levels during development was mirrored in the preferred usage of C- over U-ending Q codons in the genes most highly expressed during these developmental stages. That is, when Q levels were high, C-ending codons were preferentially used at conserved sites, whereas U-ending codons were preferred in stages where Q-levels are lower, suggesting that high Q levels in the tRNA are beneficial for C-ending codons. This model contrasts with the above described effect of Q on translation, which posits an advantage for U-ending codons in the presence of Q-containing tRNAs.

A mathematical model was developed to explain this unexpected outcome, and it illustrates how Q modification can reverse relative codon accuracy [101]. In this model, even though the U-ending codon is read more poorly by the correct non-Q-modified tRNA, it nonetheless is more accurate, because the competition with the wrong tRNA is even weaker. In other words, increased Q-levels of some tRNAs can result in more misreading of near-cognate codons, and such codons therefore are evolutionarily selected against when Q-levels are high. Thus, there is a trade-off between the evolutionary selection regarding codon choice both for increased speed and increased accuracy, which can be in conflict with each other, and coding in the genome accounts for the combined effects on codon usage in order for the organism to be optimally adjusted to its environment. Altogether, the study of Zaborske et al. [101] shows that investigation of codon usage across the genomes of related species can provide insights into the role of modifications in translation.

Such a study has not yet been performed for yeast species. With respect to the situation in *S. pombe*, where we are studying the roles of Q and m^5^C38 [12], codon usage, like in other organisms, changes with expression level of genes. For genes with low expression, there is a preference for U- over C-ending codons for the Q codons, which approx. corresponds to the overall GC content of the genome. However, above an mRNA copy number of 32 per cell, this preference was reversed towards a higher ratio of C-ending codons, indicating that translational speed, which is necessary for highly expressed genes (many of them ribosomal proteins), prefers the C-ending codons [103]. A more comprehensive investigation of codon usage at evolutionarily conserved amino acid positions in related yeast species will shed light on whether tRNA modification impacts on codon choice in these organisms.

## 6. Conclusions

The original description of Q modification of tRNAs dates back to 1972 [104], and yet, our knowledge of its effects on translation in vivo is still surprisingly limited. Studies on Dnmt2 were largely fuelled by the discovery of its tRNA methylation activity in 2006 [4], and studies of its role in translation are beginning to emerge [53,54]. The advent of ribosome profiling as a highly quantitative method for measuring translational effects opens up new avenues for the exploration of combined effects of Q and Dnmt2 on translation. For both Q and m^5^C methylation, it will be important to see whether RNAs other than tRNAs carry the modifications, especially in light of the fact that other modifications like pseudouridylation have also been discovered in mRNA [105]. It will furthermore be useful to identify the nucleases associated with the bacterial Q-related restriction-modification system, since they might give indications towards Q-specific tRNA nucleases. The availability of such enzymes as tools will also open up technical possibilities for the detection of such modifications.

Another highly important question is that of conservation of the Q-dependence of Dnmt2 in higher eukaryotes, which has broad implications for the regulation of translation in such organisms through the bioavailability of Q from environmental sources and from the microbiome. It is tantalizing to consider that codon usage in our genomes may have been subject to natural selection based on the colonization of our guts by microbes.

## Figures and Tables

**Table 1:** Overview over Dnmt2 homologs and their substrates and the presence of the transfer RNA (tRNA) guanine transglycosylase (TGT) in several model organisms.

Organism	Dnmt2 Homolog	Dnmt2 Substrate(s)	Presence of TGT
*Mus musculus*	Dnmt2	tRNA^Asp^, tRNA^Gly^, tRNA^Val^	present
*Drosophila melanogaster*	Dnmt2	tRNA^Asp^, tRNA^Gly^, tRNA^Val^	present
*Caenorhabditis elegans*	none	-	present
*Dictyostelium discoideum*	DnmA	tRNA^Asp^ (in vivo and in vitro), tRNA^Glu^ (*only* in vitro), tRNA^Gly^ (only in vitro)	present
*Schizosaccharomyces pombe*	Dnmt2/Pmt1	tRNA^Asp^ (depends on Q or Pmt1 overexpression), tRNAGlu (weak, only upon Pmt1 overexpression)	present
*Saccharomyces cerevisiae*	none	-	none
*Geobacter sulfurreducens*	Dnmt2	tRNA^Glu^	present

Pmt1: pombe methyltransferase 1; Q: queuosine.

## References

[1] Yoder J.A., Bestor T.H. (1998). A candidate mammalian DNA methyltransferase related to pmt1p of fission yeast. Hum. Mol. Genet..

[2] Hermann A., Schmitt S., Jeltsch A. (2003). The human Dnmt2 has residual DNA-(cytosine-C5) methyltransferase activity. J. Biol. Chem..

[3] Jeltsch A., Nellen W., Lyko F. (2006). Two substrates are better than one: Dual specificities for Dnmt2 methyltransferases. Trends Biochem. Sci..

[4] Goll M.G., Kirpekar F., Maggert K.A., Yoder J.A., Hsieh C.L., Zhang X., Golic K.G., Jacobsen S.E., Bestor T.H. (2006). Methylation of tRNA^Asp^ by the DNA methyltransferase homolog Dnmt2. Science.

[5] Jeltsch A., Ehrenhofer-Murray A., Jurkowski T.P., Lyko F., Reuter G., Ankri S., Nellen W., Schaefer M., Helm M. (2016). Mechanism and biological role of Dnmt2 in Nucleic Acid Methylation. RNA Biol..

[6] Shanmugam R., Aklujkar M., Schafer M., Reinhardt R., Nickel O., Reuter G., Lovley D.R., Ehrenhofer-Murray A., Nellen W., Ankri S. (2014). The Dnmt2 RNA methyltransferase homolog of *Geobacter sulfurreducens* specifically methylates tRNA-Glu. Nucleic Acids Res..

[7] Jurkowski T.P., Jeltsch A. (2011). On the evolutionary origin of eukaryotic DNA methyltransferases and Dnmt2. PLoS ONE.

[8] Bird A.W., Yu D.Y., Pray-Grant M.G., Qiu Q., Harmon K.E., Megee P.C., Grant P.A., Smith M.M., Christman M.F. (2002). Acetylation of histone H4 by Esa1 is required for DNA double-strand break repair. Nature.

[9] Antequera F., Tamame M., Villanueva J.R., Santos T. (1984). DNA methylation in the fungi. J. Biol. Chem..

[10] Becker M., Muller S., Nellen W., Jurkowski T.P., Jeltsch A., Ehrenhofer-Murray A.E. (2012). Pmt1, a Dnmt2 homolog in *Schizosaccharomyces pombe*, mediates tRNA methylation in response to nutrient signaling. Nucleic Acids Res..

[11] Pinarbasi E., Elliott J., Hornby D.P. (1996). Activation of a yeast pseudo DNA methyltransferase by deletion of a single amino acid. J. Mol. Biol..

[12] Muller M., Hartmann M., Schuster I., Bender S., Thuring K.L., Helm M., Katze J.R., Nellen W., Lyko F., Ehrenhofer-Murray A.E. (2015). Dynamic modulation of Dnmt2-dependent tRNA methylation by the micronutrient queuine. Nucleic Acids Res..

[13] Fergus C., Barnes D., Alqasem M.A., Kelly V.P. (2015). The queuine micronutrient: Charting a course from microbe to man. Nutrients.

[14] Katze J.R., Basile B., McCloskey J.A. (1982). Queuine, a modified base incorporated posttranscriptionally into eukaryotic transfer RNA: Wide distribution in nature. Science.

[15] El Yacoubi B., Bailly M., de Crecy-Lagard V. (2012). Biosynthesis and function of posttranscriptional modifications of transfer RNAs. Annu. Rev. Genet..

[16] Okada N., Noguchi S., Nishimura S., Ohgi T., Goto T., Crain P.F., McCloskey J.A. (1978). Structure determination of a nucleoside Q precursor isolated from *E. coli* tRNA: 7-(aminomethyl)-7-deazaguanosine. Nucleic Acids Res..

[17] Noguchi S., Nishimura Y., Hirota Y., Nishimura S. (1982). Isolation and characterization of an *Escherichia coli* mutant lacking tRNA-guanine transglycosylase. Function and biosynthesis of queuosine in tRNA. J. Biol. Chem..

[18] Curnow A.W., Garcia G.A. (1994). tRNA-guanine transglycosylase from *Escherichia coli*: Recognition of dimeric, unmodified tRNA^Tyr^. Biochimie.

[19] Slany R.K., Bosl M., Kersten H. (1994). Transfer and isomerization of the ribose moiety of AdoMet during the biosynthesis of queuosine tRNAs, a new unique reaction catalyzed by the QueA protein from *Escherichia coli*. Biochimie.

[20] Miles Z.D., McCarty R.M., Molnar G., Bandarian V. (2011). Discovery of epoxyqueuosine (oQ) reductase reveals parallels between halorespiration and tRNA modification. Proc. Natl. Acad. Sci. USA.

[21] Crain P.F., Sethi S.K., Katze J.R., McCloskey J.A. (1980). Structure of an amniotic fluid component, 7-(4,5-*cis*-dihydroxy-1-cyclopenten-3-ylaminomethyl)-7-deazaguanine (queuine), a substrate for tRNA: Guanine transglycosylase. J. Biol. Chem..

[22] Farkas W.R. (1980). Effect of diet on the queuosine family of tRNAs of germ-free mice. J. Biol. Chem..

[23] Kirtland G.M., Morris T.D., Moore P.H., O’Brian J.J., Edmonds C.G., McCloskey J.A., Katze J.R. (1988). Novel salvage of queuine from queuosine and absence of queuine synthesis in *Chlorella pyrenoidosa* and *Chlamydomonas reinhardtii*. J. Bacteriol..

[24] Gaur R., Bjork G.R., Tuck S., Varshney U. (2007). Diet-dependent depletion of queuosine in tRNAs in *Caenorhabditis elegans* does not lead to a developmental block. J. Biosci..

[25] Zallot R., Brochier-Armanet C., Gaston K.W., Forouhar F., Limbach P.A., Hunt J.F., de Crecy-Lagard V. (2014). Plant, animal, and fungal micronutrient queuosine is salvaged by members of the DUF2419 protein family. ACS Chem. Biol..

[26] Deshpande K.L., Seubert P.H., Tillman D.M., Farkas W.R., Katze J.R. (1996). Cloning and characterization of cDNA encoding the rabbit tRNA-guanine transglycosylase 60-kilodalton subunit. Arch. Biochem. Biophys..

[27] Suzuki T., Suzuki T. (2014). A complete landscape of post-transcriptional modifications in mammalian mitochondrial tRNAs. Nucleic Acids Res.

[28] Chen Y.C., Kelly V.P., Stachura S.V., Garcia G.A. (2010). Characterization of the human tRNA-guanine transglycosylase: Confirmation of the heterodimeric subunit structure. RNA.

[29] Jakobi S., Nguyen T.X., Debaene F., Metz A., Sanglier-Cianferani S., Reuter K., Klebe G. (2014). Hot-spot analysis to dissect the functional protein-protein interface of a tRNA-modifying enzyme. Proteins.

[30] Chen Y.C., Brooks A.F., Goodenough-Lashua D.M., Kittendorf J.D., Showalter H.D., Garcia G.A. (2011). Evolution of eukaryal tRNA-guanine transglycosylase: Insight gained from the heterocyclic substrate recognition by the wild-type and mutant human and *Escherichia coli* tRNA-guanine transglycosylases. Nucleic Acids Res..

[31] Costa A., Pais de Barros J.P., Keith G., Baranowski W., Desgres J. (2004). Determination of queuosine derivatives by reverse-phase liquid chromatography for the hypomodification study of Q-bearing tRNAs from various mammal liver cells. J. Chromatogr. B Anal. Technol. Biomed. Life Sci..

[32] Dubois D.Y., Blaise M., Becker H.D., Campanacci V., Keith G., Giege R., Cambillau C., Lapointe J., Kern D. (2004). An aminoacyl-tRNA synthetase-like protein encoded by the *Escherichia coli* yadB gene glutamylates specifically tRNA^Asp^. Proc. Natl. Acad. Sci. USA.

[33] Blaise M., Olieric V., Sauter C., Lorber B., Roy B., Karmakar S., Banerjee R., Becker H.D., Kern D. (2008). Crystal structure of glutamyl-queuosine tRNA^Asp^ synthetase complexed with l-glutamate: Structural elements mediating tRNA-independent activation of glutamate and glutamylation of tRNA^Asp^ anticodon. J. Mol. Biol..

[34] Phillips G., de Crecy-Lagard V. (2011). Biosynthesis and function of tRNA modifications in Archaea. Curr. Opin. Microbiol..

[35] Li S., Du J., Yang H., Yin J., Ding J., Zhong J. (2013). Functional and structural characterization of Dnmt2 from *Spodoptera frugiperda*. J. Mol. Cell Biol..

[36] Schulz E.C., Roth H.M., Ankri S., Ficner R. (2012). Structure analysis of *Entamoeba histolytica* Dnmt2 (EhMeth). PLoS ONE.

[37] Dong A., Yoder J.A., Zhang X., Zhou L., Bestor T.H., Cheng X. (2001). Structure of human Dnmt2, an enigmatic DNA methyltransferase homolog that displays denaturant-resistant binding to DNA. Nucleic Acids Res..

[38] Jurkowski T.P., Shanmugam R., Helm M., Jeltsch A. (2012). Mapping the tRNA binding site on the surface of human Dnmt2 methyltransferase. Biochemistry.

[39] GtRNAdb, tRNAscan-SE analysis of complete genomes. http://gtrnadb.ucsc.edu/.

[40] Varani G., McClain W.H. (2000). The G × U wobble base pair. A fundamental building block of RNA structure crucial to RNA function in diverse biological systems. EMBO Rep..

[41] Nedialkova D.D., Leidel S.A. (2015). Optimization of Codon Translation Rates via tRNA Modifications Maintains Proteome Integrity. Cell.

[42] Tuller T., Carmi A., Vestsigian K., Navon S., Dorfan Y., Zaborske J., Pan T., Dahan O., Furman I., Pilpel Y. (2010). An evolutionarily conserved mechanism for controlling the efficiency of protein translation. Cell.

[43] Rocha E.P. (2004). Codon usage bias from tRNA’s point of view: Redundancy, specialization, and efficient decoding for translation optimization. Genome Res..

[44] Sharp P.M., Bailes E., Grocock R.J., Peden J.F., Sockett R.E. (2005). Variation in the strength of selected codon usage bias among bacteria. Nucleic Acids Res..

[45] Ran W., Higgs P.G. (2010). The influence of anticodon-codon interactions and modified bases on codon usage bias in bacteria. Mol. Biol. Evol..

[46] Kramer E.B., Farabaugh P.J. (2007). The frequency of translational misreading errors in *E. coli* is largely determined by tRNA competition. RNA.

[47] Shah P., Gilchrist M.A. (2010). Effect of correlated tRNA abundances on translation errors and evolution of codon usage bias. PLoS Genet..

[48] Das G., Lyngdoh R.H. (2012). Role of wobble base pair geometry for codon degeneracy: Purine-type bases at the anticodon wobble position. J. Mol. Model..

[49] Grosjean H.J., de Henau S., Crothers D.M. (1978). On the physical basis for ambiguity in genetic coding interactions. Proc. Natl. Acad. Sci. USA.

[50] Demeshkina N., Jenner L., Westhof E., Yusupov M., Yusupova G. (2012). A new understanding of the decoding principle on the ribosome. Nature.

[51] Rozov A., Demeshkina N., Westhof E., Yusupov M., Yusupova G. (2015). Structural insights into the translational infidelity mechanism. Nat. Commun..

[52] Meier F., Suter B., Grosjean H., Keith G., Kubli E. (1985). Queuosine modification of the wobble base in tRNA^His^ influences ‘in vivo’ decoding properties. EMBO J..

[53] Shanmugam R., Fierer J., Kaiser S., Helm M., Jurkowski T.P., Jeltsch A. (2015). Cytosine methylation of tRNA-Asp by Dnmt2 has a role in translation of proteins containing poly-Asp sequences. Cell Discov..

[54] Tuorto F., Herbst F., Alerasool N., Bender S., Popp O., Federico G., Reitter S., Liebers R., Stoecklin G., Grone H.J. (2015). The tRNA methyltransferase Dnmt2 is required for accurate polypeptide synthesis during haematopoiesis. EMBO J..

[55] Manickam N., Nag N., Abbasi A., Patel K., Farabaugh P.J. (2014). Studies of translational misreading in vivo show that the ribosome very efficiently discriminates against most potential errors. RNA.

[56] Manickam N., Joshi K., Bhatt M.J., Farabaugh P.J. (2016). Effects of tRNA modification on translational accuracy depend on intrinsic codon-anticodon strength. Nucleic Acids Res..

[57] Salazar J.C., Ambrogelly A., Crain P.F., McCloskey J.A., Soll D. (2004). A truncated aminoacyl-tRNA synthetase modifies RNA. Proc. Natl. Acad. Sci. USA.

[58] Grosjean H., Westhof E. (2016). An integrated, structure- and energy-based view of the genetic code. Nucleic Acids Res..

[59] Motorin Y., Helm M. (2011). RNA nucleotide methylation. Wiley Interdiscip. Rev. RNA.

[60] Singhal R.P., Vakharia V.N. (1983). The role of queuine in the aminoacylation of mammalian aspartate transfer RNAs. Nucleic Acids Res..

[61] Satwika D., Klassen R., Meinhardt F. (2012). Anticodon nuclease encoding virus-like elements in yeast. Appl. Microbiol. Biotechnol..

[62] Kaufmann G. (2000). Anticodon nucleases. Trends Biochem. Sci..

[63] Frohloff F., Fichtner L., Jablonowski D., Breunig K.D., Schaffrath R. (2001). *Saccharomyces cerevisiae* Elongator mutations confer resistance to the *Kluyveromyces lactis* zymocin. EMBO J..

[64] Huang B., Johansson M.J., Bystrom A.S. (2005). An early step in wobble uridine tRNA modification requires the Elongator complex. RNA.

[65] Schaefer M., Pollex T., Hanna K., Tuorto F., Meusburger M., Helm M., Lyko F. (2010). RNA methylation by Dnmt2 protects transfer RNAs against stress-induced cleavage. Genes Dev..

[66] Ogawa T., Tomita K., Ueda T., Watanabe K., Uozumi T., Masaki H. (1999). A cytotoxic ribonuclease targeting specific transfer RNA anticodons. Science.

[67] Yajima S., Inoue S., Ogawa T., Nonaka T., Ohsawa K., Masaki H. (2006). Structural basis for sequence-dependent recognition of colicin E5 tRNase by mimicking the mRNA-tRNA interaction. Nucleic Acids Res..

[68] Thiaville J.J., Kellner S.M., Yuan Y., Hutinet G., Thiaville P.C., Jumpathong W., Mohapatra S., Brochier-Armanet C., Letarov A.V., Hillebrand R. (2016). Novel genomic island modifies DNA with 7-deazaguanine derivatives. Proc. Natl. Acad. Sci. USA.

[69] Wilkinson C.R., Bartlett R., Nurse P., Bird A.P. (1995). The fission yeast gene pmt1^+^ encodes a DNA methyltransferase homologue. Nucleic Acids Res..

[70] Schaefer M., Lyko F. (2010). Solving the Dnmt2 enigma. Chromosoma.

[71] Rai K., Chidester S., Zavala C.V., Manos E.J., James S.R., Karpf A.R., Jones D.A., Cairns B.R. (2007). Dnmt2 functions in the cytoplasm to promote liver, brain, and retina development in zebrafish. Genes Dev..

[72] Tuorto F., Liebers R., Musch T., Schaefer M., Hofmann S., Kellner S., Frye M., Helm M., Stoecklin G., Lyko F. (2012). RNA cytosine methylation by Dnmt2 and NSun2 promotes tRNA stability and protein synthesis. Nat. Struct. Mol. Biol..

[73] Ghanbarian H., Wagner N., Polo B., Baudouy D., Kiani J., Michiels J.F., Cuzin F., Rassoulzadegan M., Wagner K.D. (2016). Dnmt2/Trdmt1 as Mediator of RNA Polymerase II Transcriptional Activity in Cardiac Growth. PLoS ONE.

[74] Rassoulzadegan M., Grandjean V., Gounon P., Vincent S., Gillot I., Cuzin F. (2006). RNA-mediated non-mendelian inheritance of an epigenetic change in the mouse. Nature.

[75] Kiani J., Grandjean V., Liebers R., Tuorto F., Ghanbarian H., Lyko F., Cuzin F., Rassoulzadegan M. (2013). RNA-mediated epigenetic heredity requires the cytosine methyltransferase Dnmt2. PLoS Genet..

[76] Chen Q., Yan M., Cao Z., Li X., Zhang Y., Shi J., Feng G.H., Peng H., Zhang X., Zhang Y. (2016). Sperm tsRNAs contribute to intergenerational inheritance of an acquired metabolic disorder. Science.

[77] Sharma U., Conine C.C., Shea J.M., Boskovic A., Derr A.G., Bing X.Y., Belleannee C., Kucukural A., Serra R.W., Sun F. (2016). Biogenesis and function of tRNA fragments during sperm maturation and fertilization in mammals. Science.

[78] Kunert N., Marhold J., Stanke J., Stach D., Lyko F. (2003). A Dnmt2-like protein mediates DNA methylation in *Drosophila*. Development.

[79] Phalke S., Nickel O., Walluscheck D., Hortig F., Onorati M.C., Reuter G. (2009). Retrotransposon silencing and telomere integrity in somatic cells of *Drosophila* depends on the cytosine-5 methyltransferase DNMT2. Nat. Genet..

[80] Schaefer M., Lyko F. (2010). Lack of evidence for DNA methylation of Invader4 retroelements in *Drosophila* and implications for Dnmt2-mediated epigenetic regulation. Nat. Genet..

[81] Durdevic Z., Hanna K., Gold B., Pollex T., Cherry S., Lyko F., Schaefer M. (2013). Efficient RNA virus control in *Drosophila* requires the RNA methyltransferase Dnmt2. EMBO Rep..

[82] LePage D.P., Jernigan K.K., Bordenstein S.R. (2014). The relative importance of DNA methylation and Dnmt2-mediated epigenetic regulation on *Wolbachia* densities and cytoplasmic incompatibility. PeerJ.

[83] Durdevic Z., Mobin M.B., Hanna K., Lyko F., Schaefer M. (2013). The RNA methyltransferase Dnmt2 is required for efficient Dicer-2-dependent siRNA pathway activity in *Drosophila*. Cell Rep..

[84] Lin M.J., Tang L.Y., Reddy M.N., Shen C.K. (2005). DNA methyltransferase gene dDnmt2 and longevity of *Drosophila*. J. Biol. Chem..

[85] Yadlapalli S., Yamashita Y.M. (2013). Chromosome-specific nonrandom sister chromatid segregation during stem-cell division. Nature.

[86] Arya D., Kapoor S., Kapoor M. (2016). *Physcomitrella patens* DNA methyltransferase 2 is required for recovery from salt and osmotic stress. FEBS J..

[87] Ehrenhofer-Murray A.E. (2016). Humboldt-Universität zu Berlin, Berlin, Germany.

[88] Kuhlmann M., Borisova B.E., Kaller M., Larsson P., Stach D., Na J., Eichinger L., Lyko F., Ambros V., Soderbom F. (2005). Silencing of retrotransposons in *Dictyostelium* by DNA methylation and RNAi. Nucleic Acids Res..

[89] Reyniers J.P., Pleasants J.R., Wostmann B.S., Katze J.R., Farkas W.R. (1981). Administration of exogenous queuine is essential for the biosynthesis of the queuosine-containing transfer RNAs in the mouse. J. Biol. Chem..

[90] Marks T., Farkas W.R. (1997). Effects of a diet deficient in tyrosine and queuine on germfree mice. Biochem. Biophys. Res. Commun..

[91] Rakovich T., Boland C., Bernstein I., Chikwana V.M., Iwata-Reuyl D., Kelly V.P. (2011). Queuosine deficiency in eukaryotes compromises tyrosine production through increased tetrahydrobiopterin oxidation. J. Biol. Chem..

[92] Ott G., Kersten H., Nishimura S. (1982). *Dictyostelium discoideum*: A useful model system to evaluate the function of queuine and of the Q-family of tRNAs. FEBS Lett..

[93] Siard T.J., Jacobson K.B., Farkas W.R. (1991). Queuine metabolism and cadmium toxicity in *Drosophila melanogaster*. BioFactors.

[94] Durand J.M., Okada N., Tobe T., Watarai M., Fukuda I., Suzuki T., Nakata N., Komatsu K., Yoshikawa M., Sasakawa C. (1994). vacC, a virulence-associated chromosomal locus of *Shigella flexneri*, is homologous to tgt, a gene encoding tRNA-guanine transglycosylase (Tgt) of *Escherichia coli* K-12. J. Bacteriol..

[95] Durand J.M., Dagberg B., Uhlin B.E., Bjork G.R. (2000). Transfer RNA modification, temperature and DNA superhelicity have a common target in the regulatory network of the virulence of *Shigella flexneri*: The expression of the virF gene. Mol. Microbiol..

[96] Hurt J.K., Olgen S., Garcia G.A. (2007). Site-specific modification of *Shigella flexneri* virF mRNA by tRNA-guanine transglycosylase in vitro. Nucleic Acids Res..

[97] Stengl B., Meyer E.A., Heine A., Brenk R., Diederich F., Klebe G. (2007). Crystal structures of tRNA-guanine transglycosylase (Tgt) in complex with novel and potent inhibitors unravel pronounced induced-fit adaptations and suggest dimer formation upon substrate binding. J. Mol. Biol..

[98] Varghese S., Cotter M., Chevot F., Fergus C., Cunningham C., Mills K.H., Connon S.J., Southern J.M., Kelly V.P. (2016). In vivo modification of tRNA with an artificial nucleobase leads to full disease remission in an animal model of multiple sclerosis. Nucleic Acids Res..

[99] Vinayak M., Pathak C. (2009). Queuosine modification of tRNA: Its divergent role in cellular machinery. Biosci. Rep..

[100] Jacobson K.B., Farkas W.R., Katze J.R. (1981). Presence of queuine in *Drosophila melanogaster*: Correlation of free pool with queuosine content of tRNA and effect of mutations in pteridine metabolism. Nucleic Acids Res..

[101] Zaborske J.M., DuMont V.L., Wallace E.W., Pan T., Aquadro C.F., Drummond D.A. (2014). A nutrient-driven tRNA modification alters translational fidelity and genome-wide protein coding across an animal genus. PLoS Biol..

[102] White B.N., Tener G.M. (1973). Activity of a transfer RNA modifying enzyme during the development of *Drosophila* and its relationship to the su(s) locus. J. Mol. Biol..

[103] Hiraoka Y., Kawamata K., Haraguchi T., Chikashige Y. (2009). Codon usage bias is correlated with gene expression levels in the fission yeast *Schizosaccharomyces pombe*. Genes Cells.

[104] Harada F., Nishimura S. (1972). Possible anticodon sequences of tRNA^His^, tRNA^Asm^, and tRNA^Asp^ from *Escherichia coli* B. Universal presence of nucleoside Q in the first postion of the anticondons of these transfer ribonucleic acids. Biochemistry.

[105] Karijolich J., Yi C., Yu Y.T. (2015). Transcriptome-wide dynamics of RNA pseudouridylation. Nat. Rev. Mol. Cell Biol..

